# Supplementary material for: The insulin/IGF axis is critically important for controlling gene transcription in the podocyte
Source: eLife. 2026 Sep 4;14:RP107791. doi: 10.7554/eLife.107791 (PMC13544910; doi:10.7554/eLife.107791)
Supplement: Peer review history

# Editor report: eLife Assessment

**DOI:** 10.7554/eLife.107791.5.sa0

**Authors:** Weibin Zhou

**Affiliations:** Icahn School of Medicine at Mount Sinai United States

**Keywords:** Solid, Valuable

This **valuable** study used genetic and pharmacological manipulations of insulin/IGF signaling to address the role of insulin/IGF axis in the function of renal glomerular podocyte. **Solid** data are presented to demonstrate that co-inhibition of insulin/IGF signaling in podocytes led to aberrant splicing of mRNAs, which could contribute to the loss of podocytes in vitro and in vivo in mice. In light of the fact that IR/IGF-1R signaling are critically required for normal development and growth in multiple cells and organs, the lack of the assessment of developmental phenotype of podocytes in the mouse model limits the interpretation of the data.

[Editors' note: this paper was reviewed by Review Commons.]

# Referee report: Reviewer #1 (Public review)

**DOI:** 10.7554/eLife.107791.5.sa1

[Editors' note: this version has been assessed by the Reviewing Editor without further input from the original reviewers. The authors have addressed the comments raised in the previous round of review.]

Summary:

In this manuscript, the role of the insulin receptor and the insulin growth factor receptor was investigated in podocytes. Mice, where both receptors were deleted, developed glomerular dysfunction and developed proteinuria and glomerulrosclerosis over several months. Because of concerns about incomplete KO, the authors generated and studied podocyte cell lines where both receptors were deleted. Loss of both receptors was highly deleterious with greater than 50% cell death. To elucidate the mechanism of cell death, the authors performed global proteomics and found that spliceosome proteins were downregulated. They confirmed this directly by using long-read sequencing. These results suggest a novel role for insulin and IGF1R signaling in RNA splicing in podocytes.

This is primarily a descriptive study and no technical concerns are raised. The mechanism of how insulin and IGF1 signaling regulates splicing is not directly addressed but implicates potentially the phosphorylation downstream of these receptors. In the revised manuscript, it is shown that the mouse KO is incomplete potentially explaining the slow onset of renal insufficiency. Direct measurement of GFR and serial serum creatinines might also enhance our understanding of progression of disease, proteinuria is a strong sign of renal injury. An attempt to rescue the phenotype by overexpression of SF3B4 would also be useful but may be masked by defects in other spliceosome genes. As insulin and IGF are regulators of metabolism, some assessment of metabolic parameters would be an optional add-on.

Significance:

With the GLP1 agonists providing renal protection, there is great interest in understanding the role of insulin and other incretins in kidney cell biology. It is already known that Insulin and IGFR signaling play important roles in other cells of the kidney. So, there is great interest in understanding these pathways in podocytes. The major advance is that these two pathways appear to have a role in RNA metabolism.

# Referee report: Reviewer #2 (Public review)

**DOI:** 10.7554/eLife.107791.5.sa2

Summary:

In this manuscript, Coward and colleagues report on the role of insulin/IGF axis in podocyte gene transcription. They knocked out both the insulin and IGFR1 mice. Dual KO mice manifested a severe phenotype, with albuminuria, glomerulosclerosis, renal failure and death at 4-24 weeks.

Long read RNA sequencing was used to assess splicing events. Podocyte transcripts manifesting intron retention were identified. Dual knock-out podocytes manifested more transcripts with intron retention (18%) compared wild-type controls (18%), with an overlap between experiments of ~30%.

Transcript productivity was also assessed using FLAIR-mark-intron-retention software. Intron retention w seen in 18% of ciDKO podocyte transcripts compared to 14% of wild-type podocyte transcripts (P=0.004), with an overlap between experiments of ~30% (indicating the variability of results with this method). Interestingly, ciDKO podocytes showed downregulation of proteins involved in spliceosome function and RNA processing, as suggested by LC/MS and confirmed by Western blot.

Pladienolide (a spliceosome inhibitor) was cytotoxic to HeLa cells and to mouse podocytes, but no toxicity was seen in murine glomerular endothelial cells.

The manuscript is generally clear and well-written. Mouse work was approved in advance. The four figures are generally well-designed, bars/superimposed dot-plots.

Methods are generally well described.

Comments on previous version:

Coward and colleagues have done an excellent job of responding to all the reviewer comments.

# Referee report: Reviewer #4 (Public review)

**DOI:** 10.7554/eLife.107791.5.sa3

This report entitled "The insulin/IGF axis is critically important (for) controlling gene transcription in the podocyte" from Hurcombe et al is based on a mouse double knockdown of the IR and IGF1R and a parallel cultured mouse podocyte model. Insulin/IGF signaling system in mammals evolved as three gene reduplicated peptides (insulin, IGF-1, and IGF-2) and their two receptors IR and IGF1R that cross-react to variable extents with the peptides, are ubiquitously expressed, and signal through parallel pathways. The major downstream effect of insulin is to regulate glucose uptake and metabolism, while that of the IGF pathways is to regulate growth and cell cycling in part through mTORC1. The GH-IGF-1-IGF1R pathway regulates post-natal growth. IGF-2 signaling is thought to play a major role in regulating intrauterine growth and development, although IGF-2 is also present at high levels in post-natal life. Thus, one would anticipate that reducing IR/IGF1R signaling in any cell would slow growth and cell cycling by reducing growth factor and metabolic mTORC1-mediated and other processes including the splicing of RNA for protein synthesis.

# Author comment: Author response

**DOI:** 10.7554/eLife.107791.5.sa4

**Authors:** Jenny A Hurcombe, Lusyan Dayalan, Fern Barrington, Frederic Burdet, Lan Ni, Joseph Talih Coward, Paul Brinkkotter, Mark Ibberson, Martin Holzenberger, Aaron R Jeffries, Sebastian Oltean, Gavin I Welsh, Richard JM Coward

**Affiliations:** University of Bristol Bristol United Kingdom; University of Bristol Bristol United Kingdom; University of Bristol Bristol United Kingdom; SIB Swiss Institute of Bioinformatics Lausanne Switzerland; Bristol Renal, University of Bristol Bristol United Kingdom; University of Bristol Bristol United Kingdom; University of Cologne Cologne Germany; SIB Swiss Institute of Bioinformatics Lausanne Switzerland; INSERM and Sorbonne Universities, UPMC Paris France; University of Exeter exeter United Kingdom; University of Exeter Exeter United Kingdom; University of Bristol Bristol United Kingdom; University of Bristol Bristol United Kingdom

The following is the authors’ response to the previous reviews

**Public Reviews:**

**Reviewer #1 (Public review):**

Summary:

In this manuscript, the role of the insulin receptor and the insulin growth factor receptor was investigated in podocytes. Mice, where both receptors were deleted, developed glomerular dysfunction and developed proteinuria and glomerulrosclerosis over several months. Because of concerns about incomplete KO, the authors generated and studied podocyte cell lines where both receptors were deleted. Loss of both receptors was highly deleterious with greater than 50% cell death. To elucidate the mechanism of cell death, the authors performed global proteomics and found that spliceosome proteins were downregulated. They confirmed this directly by using long-read sequencing. These results suggest a novel role for insulin and IGF1R signaling in RNA splicing in podocytes.

This is primarily a descriptive study and no technical concerns are raised. The mechanism of how insulin and IGF1 signaling regulates splicing is not directly addressed but implicates potentially the phosphorylation downstream of these receptors. In the revised manuscript, it is shown that the mouse KO is incomplete potentially explaining the slow onset of renal insufficiency. Direct measurement of GFR and serial serum creatinines might also enhance our understanding of progression of disease, proteinuria is a strong sign of renal injury. An attempt to rescue the phenotype by overexpression of SF3B4 would also be useful but may be masked by defects in other spliceosome genes. As insulin and IGF are regulators of metabolism, some assessment of metabolic parameters would be an optional add-on.

Significance:

With the GLP1 agonists providing renal protection, there is great interest in understanding the role of insulin and other incretins in kidney cell biology. It is already known that Insulin and IGFR signaling play important roles in other cells of the kidney. So, there is great interest in understanding these pathways in podocytes. The major advance is that these two pathways appear to have a role in RNA metabolism.

Latest comments:

The new reviewer raised two major points, whether the KO effect on splicing is specific to IGF1 and whether the interpretation could be developmental rather than due to splicing. The reviewer raises some important issues but the evidence to suggest that this is specific is data in the literature that IR/IGF signaling is already known to regulate splicing and that splicing defects were not detected in other models that they have analyzed. I agree with the reviewer (and authors) that the incomplete floxing of the genes is a major complication. The point that there could be a developmental defect with mice being born with fewer podocytes and perhaps the authors should caveat this point. The fact that they mice are born with normal function, that renal function can be maintained with up to 80% loss of podocytes suggest that they are likely born with a good number of podocytes and the dysfunction that occurs at 6 months is due to a process, induced by the loss of IR/IGF signaling that is detrimental to the podocyte.

Thank you for these insightful comments. We fully acknowledge that the mouse model will not have had full insulin receptor and IGF1R knockdown and that this is likely the reason it took time to develop and not give a prominent early phenotype. We agree with this reviewer and new reviewer 4 that if the model had facilitated near complete IR and IGF1R knockdown then likely a significant neonatal / embryonic phenotype would have been obvious. We considered using an inducible mouse model to allow normal development before cre-excision but our experience is that the CreER and RtTA-tet-on-cre system is less good at excising genes and hence did not pursue this (we show evidence of reduced excision with an inducible system in supplementary Figure 1D using a reporter mouse system [this was included in a previous response to the reviewers only]). This was rationale for making the immortalised podocyte floxed IR and IGF1R cell line to ensure near complete knockdown. This, not surprisingly, was highly detrimental. We then looked mechanistically for pathways (using agnostic proteomics and phospho-proteomics) and found spliceosomal involvement. From our studies we think this was also involved in our mouse model as SF3B4 was found to be significantly down regulated in the podocytes of double receptor knockdown transgenic mice (Figure 3F).

**Reviewer #2 (Public review):**

Summary:

In this manuscript, submitted to Review Commons (journal agnostic), Coward and colleagues report on the role of insulin/IGF axis in podocyte gene transcription. They knocked out both the insulin and IGFR1 mice. Dual KO mice manifested a severe phenotype, with albuminuria, glomerulosclerosis, renal failure and death at 4-24 weeks.

Long read RNA sequencing was used to assess splicing events. Podocyte transcripts manifesting intron retention were identified. Dual knock-out podocytes manifested more transcripts with intron retention (18%) compared wild-type controls (18%), with an overlap between experiments of ~30%.

Transcript productivity was also assessed using FLAIR-mark-intron-retention software. Intron retention w seen in 18% of ciDKO podocyte transcripts compared to 14% of wild-type podocyte transcripts (P=0.004), with an overlap between experiments of ~30% (indicating the variability of results with this method). Interestingly, ciDKO podocytes showed downregulation of proteins involved in spliceosome function and RNA processing, as suggested by LC/MS and confirmed by Western blot.

Pladienolide (a spliceosome inhibitor) was cytotoxic to HeLa cells and to mouse podocytes but no toxicity was seen in murine glomerular endothelial cells.

The manuscript is generally clear and well-written. Mouse work was approved in advance. The four figures are generally well-designed, bars/superimposed dot-plots.

Methods are generally well described.

Comments on previous version:

Coward and colleagues have done an excellent job of responding to all the reviewer comments.

Thank you.

**Reviewer #4 (Public review):**

Summary and background:

This report entitled "The insulin/IGF axis is critically important (for) controlling gene transcription in the podocyte" from Hurcombe et al is based on a mouse double knockdown of the IR and IGF1R and a parallel cultured mouse podocyte model. Insulin/IGF signaling system in mammals evolved as three gene reduplicated peptides (insulin, IGF-1, and IGF-2) and their two receptors IR and IGF1R that cross-react to variable extents with the peptides, are ubiquitously expressed, and signal through parallel pathways. The major downstream effect of insulin is to regulate glucose uptake and metabolism, while that of the IGF pathways is to regulate growth and cell cycling in part through mTORC1. The GH-IGF-1-IGF1R pathway regulates post-natal growth. IGF-2 signaling is thought to play a major role in regulating intrauterine growth and development, although IGF-2 is also present at high levels in post-natal life. Thus, one would anticipate that reducing IR/IGF1R signaling in any cell would slow growth and cell cycling by reducing growth factor and metabolic mTORC1-mediated and other processes including the splicing of RNA for protein synthesis.

Thank you for this clear overview. Of note the podocyte is a terminally differentiated cell so the growth / cell cycling elements may be different from more proliferative cell types in relation IR/IGF1R mediated signalling.

Comments on revised version:

The second sentence of the Summary reads "This study sought to elucidate the compound role of the insulin/IGF1 axis in podocytes using transgenic mice and cell culture models deficient in both receptors." The study design and rationale for the proteosome analysis described is predicated on the finding that podocyte-specific knockdown of the IR/IGF-1R in mice is associated with development of proteinuria and reduced eGFR by 20months of life. Since the IR/IGF-1R are critically required for normal development and growth of all cells and organs, the obvious explanation for the observation would be that the model system results in defective podocyte development and deployment (caused by reduced IR/IGF-1) that, in turn, causes subsequent development of proteinuria and glomerulosclerosis (that may be much less dependent on a normal level of IR/IGF-1R expression). Thus, the experimental design does not allow a distinction between podocyte development and steady state function which are different biologic processes. The data provided does not examine podocyte status immediately after birth to confirm that podocyte number and size and structure is normal in mice that subsequently develop proteinuria and glomerulosclerosis. The response to the reviewer suggests that since this would require additional mice it has not been undertaken in order to reduce animal usage. This is not a valid argument, particularly when the investigators have not even used state-of-the-art methods to measure podocyte number, size and density in adult mice, key parameters that would be required to interpret their data. Counting podocyte nuclear number in glomerular cross-sections is simply an inadequate method, even if it is used and reported in other journals, and particularly where the examples given to justify its use can hardly be viewed as representing first rate science.

Thank you for these comments. As discussed above we agree that the mouse model was not optimal as despite using a good cre driver we did not consistently knock down both receptors. It was the reason that we made the IR/IGF1R knockdown cell line. Importantly we found with both receptors >80% knocked down that this was highly detrimental and evidence that spliceosomal dysfunction was prominent. Thank you for the comment about methodology of assessing podocyte number which we and other investigators use.

If the absence of studies that would answer the above questions, the investigators should add a sentence to the Discussion dealing with study limitations as follows. "The study design does not allow us to determine whether the primary effect of reduced IR/IGF-1R expression on the phenotype is during in utero and post-natal podocyte development and deployment, during periods of rapid growth when IGF-1 levels are highest, in steady state adult podocytes, or under all of the above conditions".

Thank you. We have added a section describing that we did not investigate the embryonic neonatal early phenotype for more subtle changes in our model. We have also added a sentence saying we would have liked to have used an inducible model but the cre driven excision is less than constitutional driver and we think would have shown either a very mild or no phenotype due to minimal excision.
